# Supplementary material for: Interrogating site dependent kinetics over SiO2-supported Pt nanoparticles
Source: Nat Commun. 2024 Mar 7;15:2074. doi: 10.1038/s41467-024-46496-1 (PMC10920675; doi:10.1038/s41467-024-46496-1)
Supplement: Supplementary file 1 — Supplementary Information [file 41467_2024_46496_MOESM1_ESM.pdf]

Interrogating Site Dependent Kinetics over SiO_2-Supported Pt Nanoparticles

Taek-Seung Kim, Christopher R. O'Connor and Christian Reece*

Rowland Institute at Harvard, Harvard University, Cambridge, MA 02142
*Corresponding author: christianreece@fas.harvard.edu

Supplementary Information

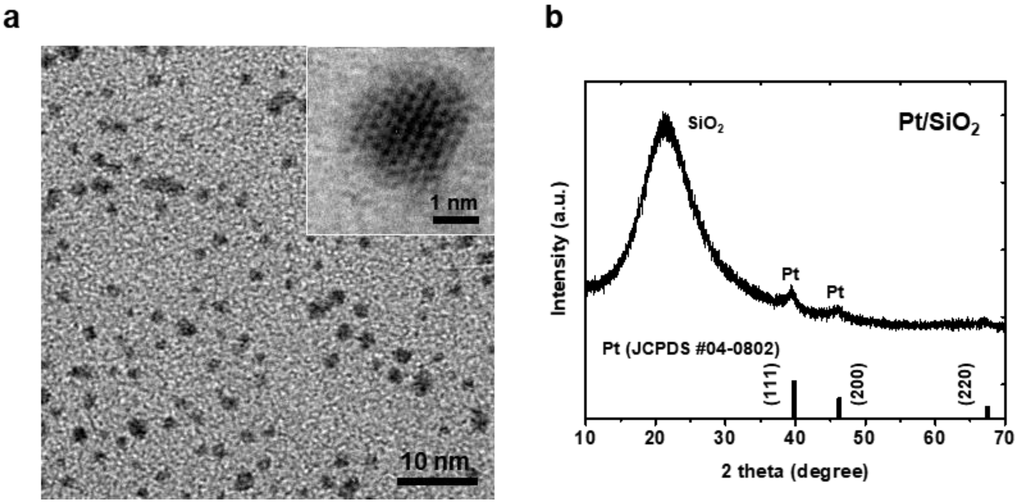

Supplementary Figure 1. Characterization of as synthesized 2nm Pt/SiO_2 catalyst a, Representative TEM image with high resolution TEM image (inset) of as-synthesized Pt nanoparticles. **b**, XRD pattern of Pt/SiO_2 catalyst with reference Pt (JCPDS #04-0802).

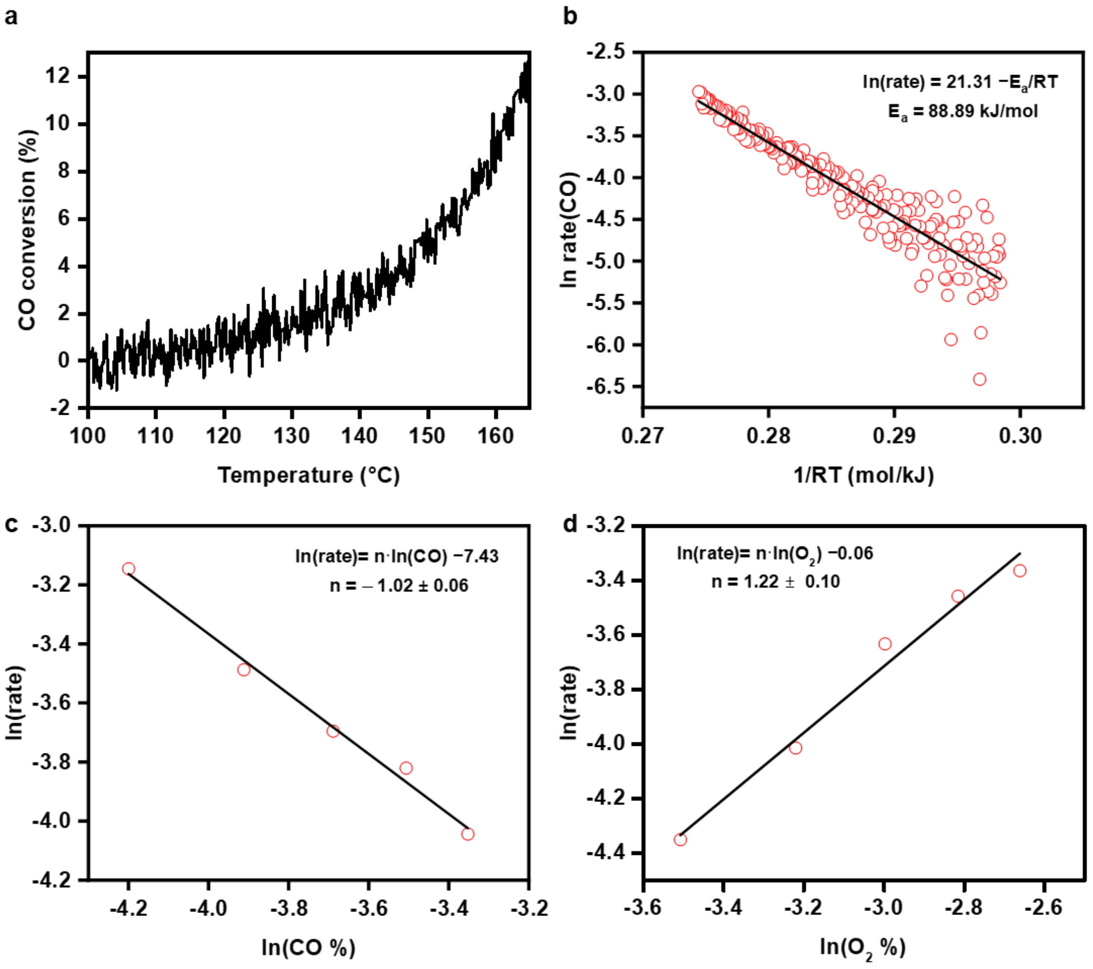

Supplementary Figure 2. Kinetics of CO oxidation over 2nm Pt/SiO_2 catalyst a, Temperature programmed reaction spectra for CO oxidation over 2 nm Pt/SiO_2 from the rate of CO consumption using a heating rate of 5 °C/min (100 ml/min total flow; 2.5%CO, 5%O_2) from 100 to 170 °C. **b**, Determination of the apparent activation energy from 130 to 160 °C of data in (a). **c,d**, Determination of the reaction order in (c) CO (1.5−3.5%CO, 5 %O_2) and (d) O_2 (2.5%CO, 3−7%O_2) from the rate of CO_2 production at 160 °C. The rate of CO_2 production was used for determining the reaction orders because of the higher sensitivity to CO_2 production than CO consumption at low conversions. The total conversion of reactants relative to the entire gas stream is sufficiently low that any change in relative concentration due to the non-stoichiometric reaction is within the noise of the mass spectrometer signal.

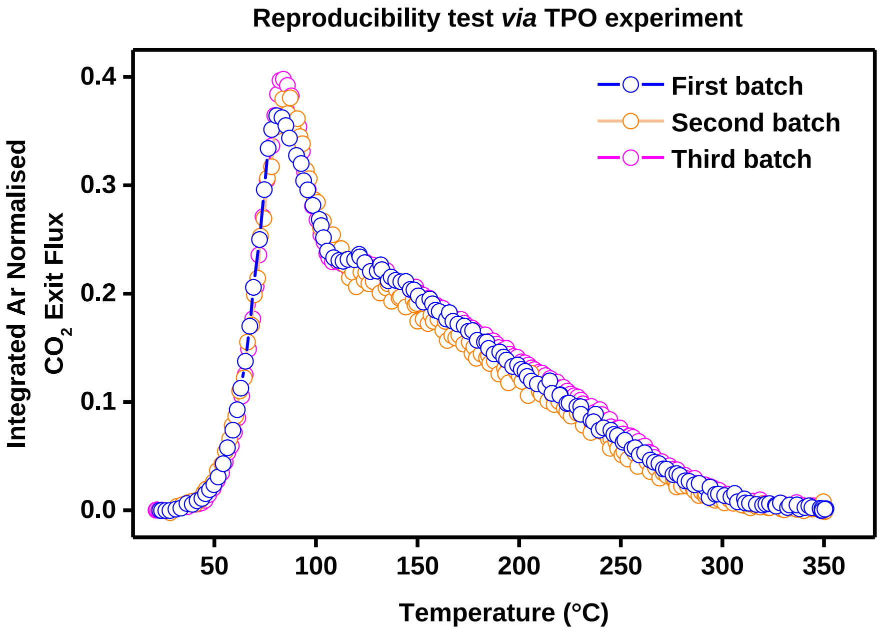

Supplementary Figure 3. Reproducibility testing of 2nm Pt/.SiO_2 catalyst. Integrated Ar normalised exit flux of m/z = 44 (CO_2) during temperature programmed oxidation (TPO) experiment on the CO^*-covered Pt/SiO_2 catalyst where CO^* was preadsorbed at 25 °C. Then O_2 was repeatedly pulsed over the catalyst while being linearly heated to 350 °C at 8 °C/min. The weights of the Pt/SiO_2 catalyst for each batch are 5.40, 4.88, and 5.38 mg.

I. Generation of TAP adsorption/reaction models using Multi-Zone TAP Reactor Theory

The analytical solutions for the TAP adsorption/reaction models are generated using Multi-Zone TAP Reactor Theory[1,2] with the catalyst zone being approximated as a thin-zone sandwiched between two layers of inert packing. MZTRT has been described extensively in a series of publications[1–4], but it is summarised here for clarity. The TAP microreactor is split into zones, each of which can have its own diffusion and/or reaction properties. The most common format of the TAP system is the Thin-Zone TAP reactor[5] where a thin layer of catalyst is placed at the centre of the packed bed with a layer of inert packing on either side. All functions generated from MZTRT are in the Laplace domain and are as a function of the Laplace variable s, but they can be approximated in the Fourier domain[6,7] which can be converted to the time domain *via* an Inverse Fast Fourier Transform algorithm.

Each zone in the TAP reactor is represented by a $2n_g \times 2n_g$ transfer matrix, where n_g is the number of gases to be modelled. The transfer matrices are then multiplied together to generate the transfer matrix for the TAP reactor:

$$\begin{pmatrix} P_{11}(s) & P_{12}(s) \\ P_{21}(s) & P_{22}(s) \end{pmatrix} = M_1(s)M_2(s)\dots M_N(s) \qquad (1)$$

where M_N is the $2n_g \times 2n_g$ matrix for each zone N, and P_{nn} is a $n_g \times n_g$ matrix that contains information about the concentration and flux of gas in the reactor. Specifically, the matrix P_{22} contains the information about the flux of the gases at the exit of a reactor during the pulse such that:

$$P_{22}(s) = \begin{pmatrix} F_{exit}^{1}(s) & \sim \\ F_{exit}^{n}(s) & \sim \end{pmatrix} \qquad (2)$$

where F_{exit}^{n} is the flux of gas leaving the reactor exit for gas n. The application of MZTRT may appear to be complex, but through the following method it becomes feasible to build a transfer matrix M_n for any linear series of reactions. The example outlined here is for a simple reversible adsorption + reaction model where a single reactant can reversibly adsorb to a surface and convert into a single product. This model contains 2 gases, 1 surface species, and three rate constants k_a, k_d, and k_r.

$$A \underset{k_d}{\overset{k_a}{\rightleftharpoons}} A^* \overset{k_r}{\rightarrow} B \qquad (3)$$

First, the $n_g \times n_g$ transport matrix is assembled for each zone:

$$D_N = \begin{pmatrix} D_{e,r} & \\ & D_{e,p} \end{pmatrix} \quad (4)$$

For a given zone N the transport matrix D_N is assembled as a sparse matrix containing the diffusivities for the gases $D_{e,n}$ for that given zone on the diagonal. The kinetic matrix is then assembled. First, a $n_g \times n_g$ matrix containing the rate constants for the gases as a function of the gases is assembled, which for the reversible adsorption + reaction model would be:

$$R_{gg} = \begin{pmatrix} -k_a & 0 \\ 0 & 0 \end{pmatrix} \quad (5)$$

Then, a $n_g \times n_s$ matrix is assembled where n_s is the number of surface species where the rate of the gases as is represented as a function of the surface species:

$$R_{gs} = \begin{pmatrix} k_d \\ k_r \end{pmatrix} \quad (6)$$

Then, a $n_s \times n_g$ matrix is assembled where the rate of the surface species is represented as a function of the gaseous species:

$$R_{sg} = \begin{pmatrix} k_a & 0 \end{pmatrix} \quad (7)$$

Then, a $n_s \times n_s$ matrix is assembled where the rate of the surface species is represented as a function of the surface species:

$$R_{ss} = (-k_d - k_r) \quad (8)$$

The reactivity matrix is then assembled using the following:

$$R = -R_{gg} - R_{gs}\left(sI_{(n_s)} - R_{ss}\right)^{-1} R_{sg} \quad (9)$$

where $I_{(n_s)}$ is an identity matrix of size n_s. Next, the kinetic matrix for each zone is assembled *via* the following:

$$K_N = sI_{(n_g)}\varepsilon_{b,N} + R \quad (10)$$

If zone N is non-reactive (i.e., an inert zone) the matrix R is set to zero. Once the kinetic matrix has been assembled, a $2n_g \times 2n_g$ transport matrix can be assembled where:

$$T_N = \begin{pmatrix} D_N^{-1}K_N & 0_{n_g} \\ 0_{n_g} & D_N^{-1}K_N \end{pmatrix} L_N^2 \tag{11}$$

where 0_{n_g} is a zero matrix of size $n_g \times n_g$ and L is the length of the zone N. Finally, the transfer matrix for a given zone can be calculated using:

$$M_N = \cosh\sqrt{T_N} + \begin{pmatrix} 0_{n_g} & D_N^{-1} \\ K_N & 0_{n_g} \end{pmatrix} \left(\frac{\sinh\sqrt{T_N}}{\sqrt{T_N}} \right) L \tag{12}$$

For a Thin-Zone TAP reactor, the transfer matrix for the catalyst zone contains no transport, and as such can be calculated using:

$$M_{TZ} = I_{(2n_g)} \begin{pmatrix} 0_{n_g} & 0_{n_g} \\ R & 0_{n_g} \end{pmatrix} \tag{13}$$

The analytical solution for a reversible adsorption + reaction case can be generated using the Symbolic Math Toolbox and the following MATLAB code.

```
% Generates Reversible adsorption / reaction analytical
solution.
% s     : Laplace Variable
% ka    : Adsorption rate constant
% kd    : Desorption rate constant
% kr    : Reaction rate constant
% eb    : Voidages (zones 1, 2, and 3 respectively)
% L     : Lengths (zones 1, 2, and 3 respectively)
% De    : Diffusivities of reactant (zones 1, 2, and 3
respectively)
% Dp    : Diffusivities of product (zones 1, 2, and 3
respectively)

clear
syms s ka kd kr eb1 eb2 eb3...
    L1 L2 L3 De1 De2 De3 Dp1 Dp2 Dp3

% Diffusivity matrix [nG x nG]
g1    = [De1,Dp1]; % Zone 1 diffusivities
g2    = [De2,Dp2]; % Zone 2 diffusivities
g3    = [De3,Dp3]; % Zone 3 diffusivities
D1    = eye(length(g1)).*g1; % Diffusivity Matrix 1
D2    = eye(length(g2)).*g2; % Diffusivity Matrix 2
D3    = eye(length(g3)).*g3; % Diffusivity Matrix 3

% G = Gas S = Surface
% Rate of G as a function of G [nG x nG]
Rgg = [-ka,0;...
```

```
        0   ,0];
% Rate of G as a function of S [nG x nS]
Rgs = [+kd;
        +kr];
% Rate of S as a function of G [nS x nG]
Rsg = [+ka, 0];
% Rate of S as a function of S [nS x nS]
Rss = [-kd-kr];

% Reactivity Matrix
R   = - Rgg - Rgs*inv(s*eye(length(Rss)) - Rss)*Rsg;

% Kinetic Matrix
Ks1           = eye(length(Rgg))*s*eb1;
Ks2           = eye(length(Rgg))*s*eb2+R;
Ks3           = eye(length(Rgg))*s*eb3;

% Transport Matrix
T1            = [inv(D1)*Ks1,zeros(length(D1));...
    zeros(length(D1)),Ks1*inv(D1)]*L1^2;
T2            = [inv(D2)*Ks2,zeros(length(D2));...
    zeros(length(D2)),Ks2*inv(D2)]*L2^2;
T3            = [inv(D3)*Ks3,zeros(length(D3));...
    zeros(length(D3)),Ks3*inv(D3)]*L3^2;

% Matrix for Z1
Mk1          = funm(sqrtm(T1),@cosh)+...

[zeros(2),inv(D1);Ks1,zeros(2)]*L1*(funm(sqrtm(T1),@sinh)/sqrt
m(T1));
% Matrix for Z2
Mk2          = funm(sqrtm(T2),@cosh)+...

[zeros(2),inv(D2);Ks2,zeros(2)]*L2*(funm(sqrtm(T2),@sinh)/sqrt
m(T2));
% Matrix for Z3
Mk3          = funm(sqrtm(T3),@cosh)+...

[zeros(2),inv(D3);Ks3,zeros(2)]*L3*(funm(sqrtm(T3),@sinh)/sqrt
m(T3));

% MZTRT Matrix
G      = Mk1*Mk2*Mk3;

% Exit Flux
P22    = inv(G(3:4,3:4)); %P22
G1  = P22(1,1); % Exit flux of reactant
G2  = P22(2,1); % Exit flux of product
```

II. Confirmation of the Three-pathway CO oxidation model

To simulate TPO and O_2 titration experiment experiments a number of MZTRT models (see section: Generation of TAP adsorption/reaction models using Multi-Zone TAP Reactor Theory) were built with varying complexity. These contain either a single irreversible adsorption/reaction site, an irreversible adsorption/reaction site + an irreversible adsorption site, two irreversible adsorption/reaction sites, and finally two irreversible adsorption/reaction sites + an irreversible adsorption site. It was found that only the three-pathway CO oxidation model was able to accurately recreate both the shape and magnitude of the exit flux curves (Supplementary Fig. 4). A model containing three irreversible adsorption/reaction + irreversible adsorption sites was also tested, and no increase in the quality of fit between the model and the experiment was detected. However, the 95% confidence intervals for the underlying kinetic processes ($k_1 – k_4$) were found to be extremely large (Supplementary Fig. 5). As such, we feel that it is highly likely that only two pathways for reaction of O_2 with preabsorbed CO exist and that under these conditions O^* is not mobile and so can irreversibly adsorb and no longer react.

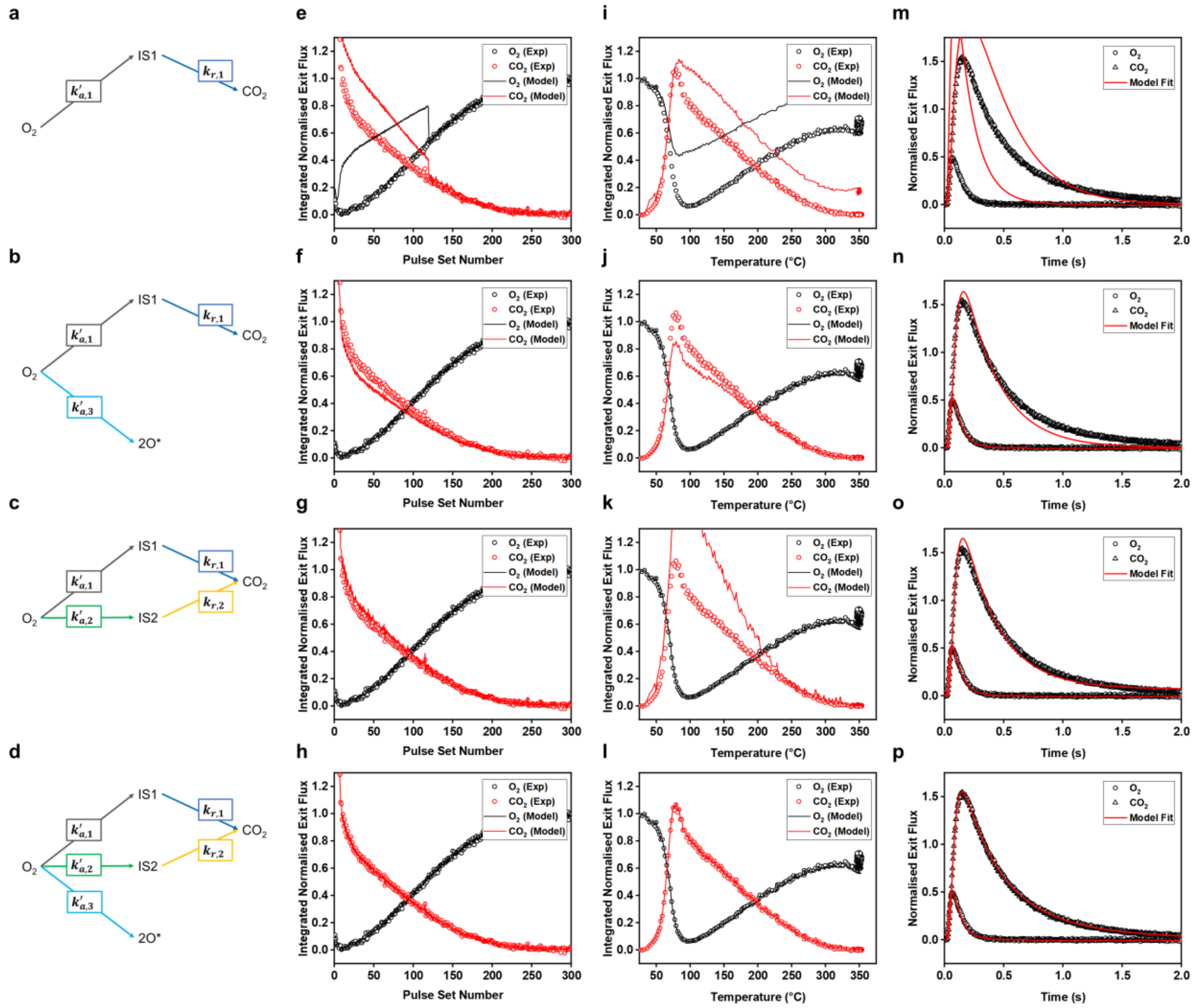

Supplementary Figure 4. Comparison of four different models used to simulated pulse experiments. a–d, The four different models used. **e–l**, Experimentally measured and model fitted integrated normalised exit flux curves during isothermal O_2 titration (**e–h**) and TPO (**i–l**) experiments. **m–p**, Experimentally measured and model exit flux curves at 100 °C in TPO

experiment. For the isothermal O_2 titration experiment, CO was pre-absorbed at 100 °C and O_2 was repeatedly pulsed over the catalyst at 100 °C. For the TPO experiment, CO was preabsorbed at 25 °C and O_2 was repeatedly pulsed over the catalyst while being linearly heated to 350 °C. Only the model containing two irreversible adsorption/reaction + irreversible adsorption sites was able to accurately recreate the TPO experiment.

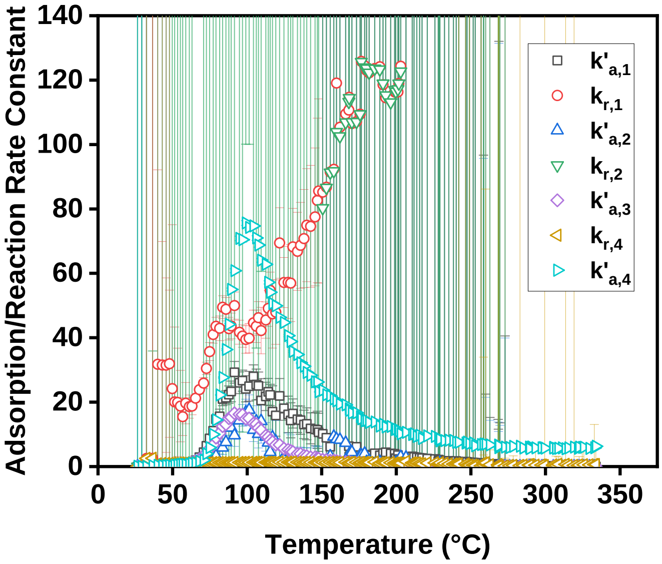

Supplementary Figure 5. Kinetic coefficients and 95% confidence intervals calculated from curve fitting to the three adsorption/reaction + one irreversible adsorption model. Open circles represent calculated rate constants, with bars representing the 95% confidence intervals for those rate constants.

Supplementary Table 1. DRIFTS ν(C−O) frequency peak maxima for CO adsorption from 35 to 350 °C.

Temperature (°C)	(1) − Linear WC (cm^{-1})	(2) − Linear UC (cm^{-1})	(3) – Multi-Bonded (cm^{-1})
35	2075	2042	1805
100	2070	2029	1782
200	2065	1989	1742
350	2052	1970	1701

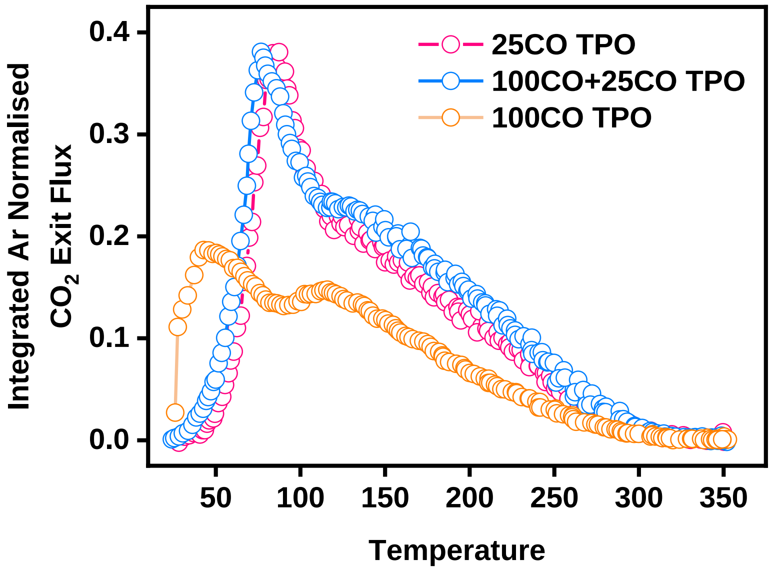

Supplementary Figure 6. TPO Renaturation Experiments. TPO experiments of 25CO, 100CO, and 100CO + 25CO. For the 100CO + 25CO TPO data, the TPO experiment was performed where CO was firstly preadsorbed at 100 °C, and then CO* was saturated at 25 °C.

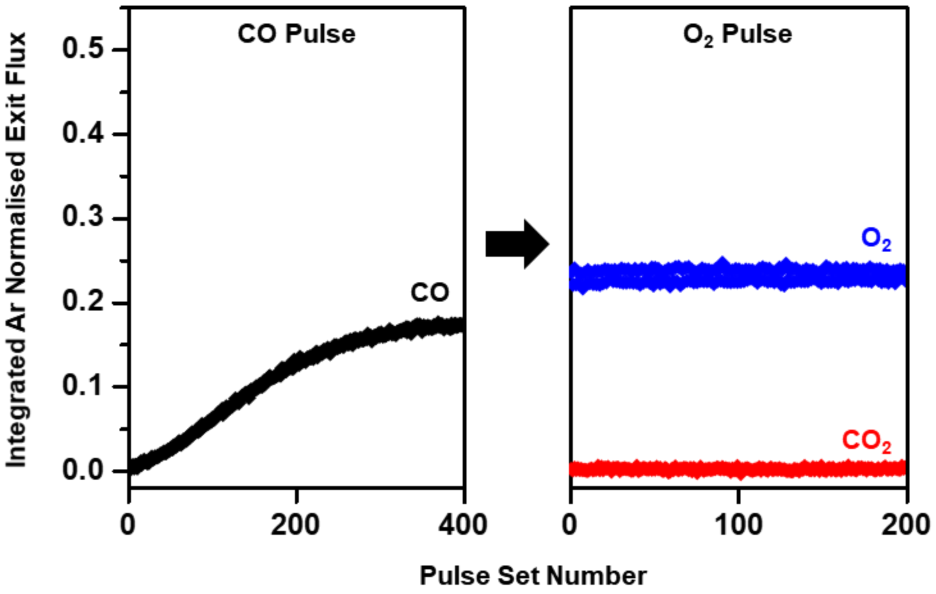

Supplementary Figure 7. Room temperature activity experiments Gas switching experiment at 25 °C on the pristine Pt/SiO_2 catalyst. CO* was pre-adsorbed at 25 °C over pristine Pt/SiO_2 catalyst (left), followed by injection of the oxygen pulses over the CO*-covered Pt/SiO_2 catalyst (right).

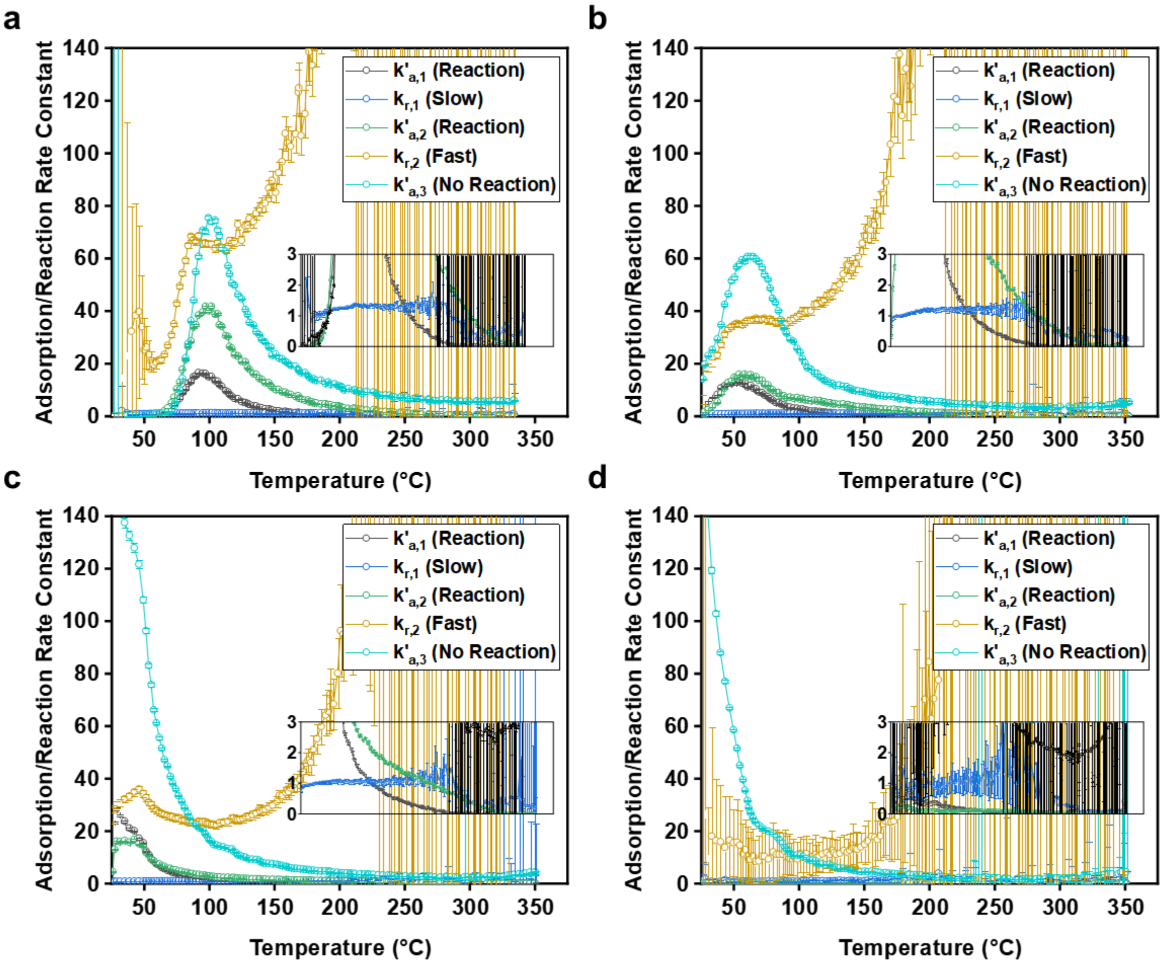

Supplementary Figure 8. Rate constants calculated from model fitting with 95% confidence intervals included for TPO experiments where CO was preadsorbed at (**a**) 25, (**b**) 100, (**c**) 200, and (**d**) 350 °C. Inset shows small but non-zero value for $k_{r,1}$.

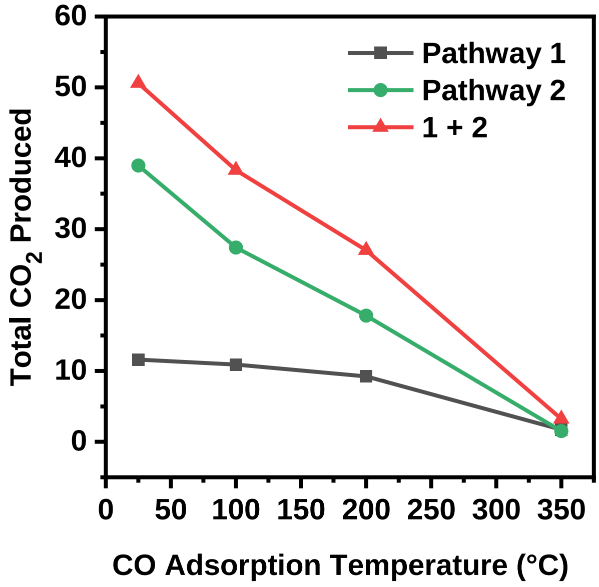

Supplementary Figure 9. The total amount of CO_2 produced by each pathway during the TPO experiments as calculated using the kinetic model. Total amount of CO_2 produced during TPO experiments where CO was adsorbed at 25, 100, 200, and 350 °C.

III. Quantification evaluation for CO adsorption sites *via* DRIFTS measurements

A quantitative evaluation of the relative population of CO adsorption sites as a function of adsorption temperature was performed by peak deconvolution, integration, and normalization by extinction coefficients. The deconvolution of linear CO and multi-bound CO is trivial due to the large frequency separation between the vibrational features (> 200 cm^{-1}). However, the deconvolution of linear well-coordinated (WC) and under-coordinated (UC) sites is challenging due to the small frequency separation between the vibrational features (~50 cm^{-1}) and the asymmetry of the linear UC feature. The extended asymmetry of the linear features originates from numerous possible under-coordinated Pt environments (*e.g.,* edge atom, corner atom, adatom) where decreasing Pt coordination number has been correlated with a linear downshift in frequency[8–10]. The deconvolution of the linear WC and UC peaks was performed by fitting a dominant linear WC peak with a high frequency shoulder using the high frequency side of the linear WC feature and attributing the residual features from the spectra to an asymmetric linear UC peak (Supplementary Fig. 10). A traditional peak fitting routine[11,12] for quantitative analysis using Gaussian, Lorentzian or Voigt peaks could not yield a unique solution that adequately fit the data. The absolute intensity of linear WC and UC features varied with the chosen method but the trend of relative changes of linear WC, UC and multi-bound features with adsorption temperature was consistent for all methods. Next, the deconvoluted features were integrated as absorbance intensity. Quantitative analysis of DRIFTS spectra can be prone to errors because adsorbate concentration is truly neither proportional to absorbance intensity nor Kubelka-Munk[13,14] (KM) intensity but can be nearly proportional to one intensity unit depending on the relative reflectivity of the DRIFTS features[15]. For future investigations, integration in Matyshak-Krylov[16] intensity is most appropriate for quantitative DRIFTS but requires non-traditional single scan FTIR measurements[15]. The relative reflectivity of our DRIFTS features demonstrate the integration of absorbance intensity is nearly proportional to concentration while Kubelka-Munk is not appropriate (Supplementary Fig. 11)[15]. Lastly, the integral of the linear UC and multi-bound features were divided by Pt environment specific extinction coefficients to convert from peak areas to adsorbate concentrations. The relative extinction coefficient of linear UC to linear WC Pt has been determined to be ~ 2.7 from FTIR measurements on Pt single crystals[17,18]. We estimated the relative extinction coefficient of multi-bound to linear WC Pt to be 0.43 by integration of the linear WC and bridge-bound intensities of a c(4 × 2) ordering of CO on Pt(111)[19]. The utilization of relative extinction coefficients for the quantitative evaluation of the DRIFT spectra only allows for analysis of relative amounts of CO adsorbed at distinct Pt environments.

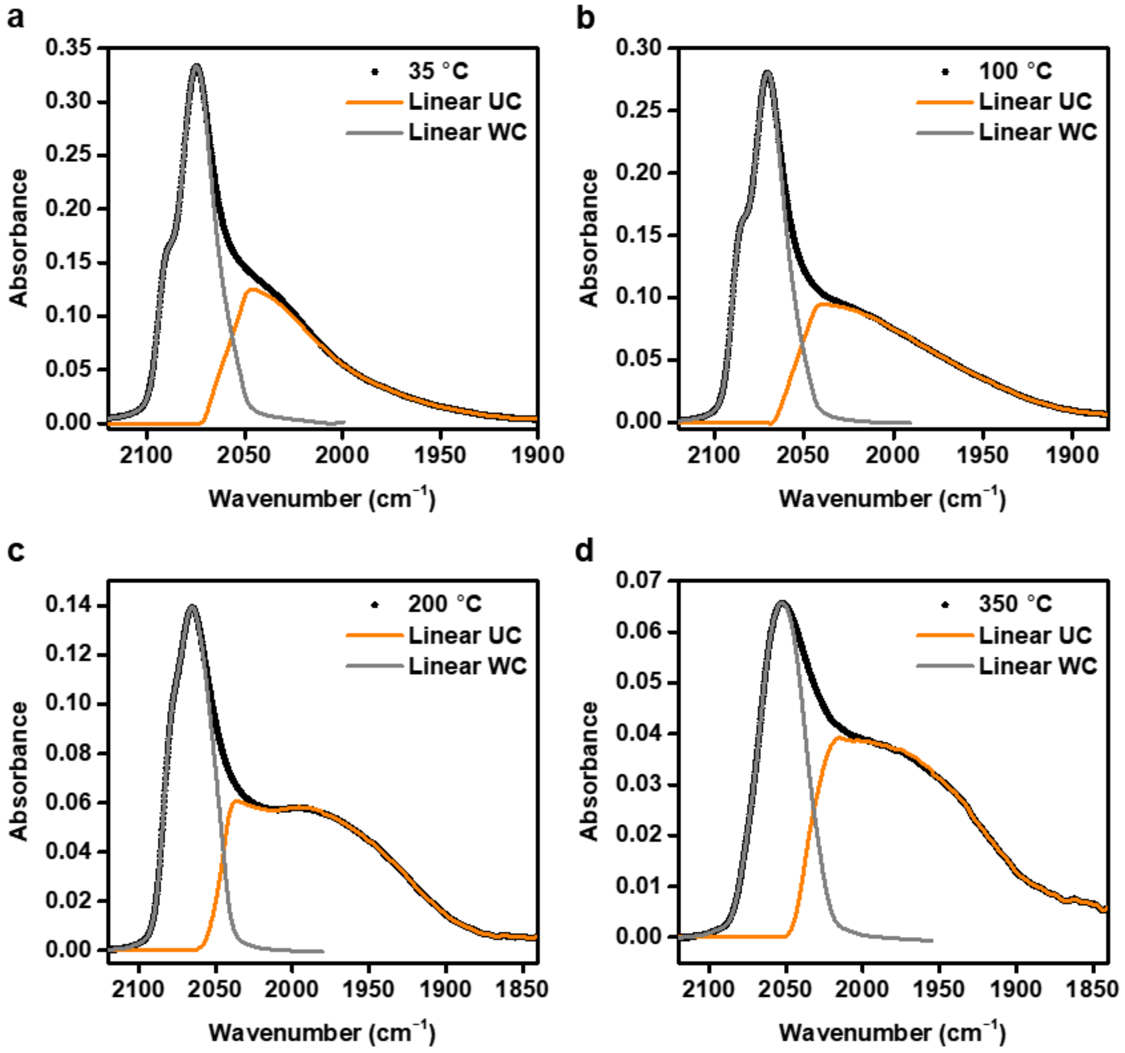

Supplementary Figure 10. DRIFTS spectral deconvolution of linear WC and linear UC features for CO adsorption. Adsorption temperatures of (**a**) 35, (**b**) 100, (**c**) 200, and (**d**) 350 °C on 2 nm Pt/SiO_2 catalyst. The deconvolution of the linear WC and UC peaks was performed by fitting a dominant linear WC peak with a high frequency shoulder for dense CO using the high frequency side of the linear WC feature. The residual features from the experimental spectra subtracted by the WC peak were attributed to an asymmetric linear UC peak. A linear UC peak with an asymmetry towards the low frequency side is physically justified because there are numerous possible under-coordinated Pt environments (*e.g.*, edge atom, corner atom, and adatom) where decreasing Pt coordination number has been correlated with a linear downshift in frequency[8–10]. A highly asymmetric linear UC peak is in reasonable agreement with previous quantitative DRIFTS investigations.

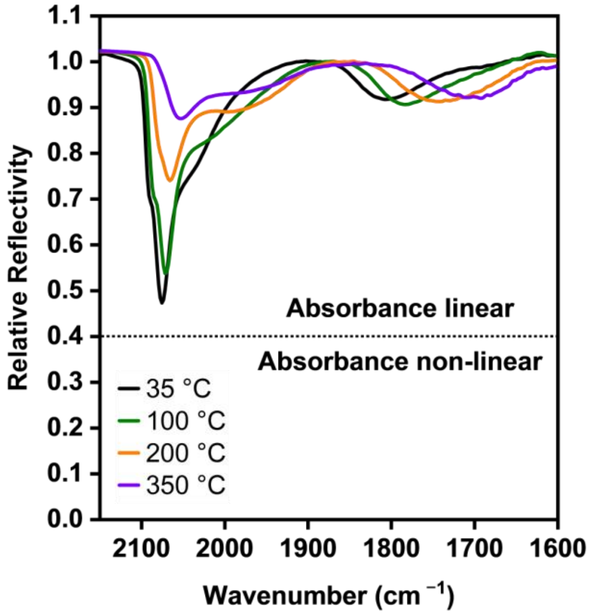

Supplementary Figure 11. DRIFT spectra in relative reflectivity units for CO adsorption on the CO*-covered Pt/SiO_2 catalyst. The relative reflectivity was calculated when CO* was preadsorbed at 35, 100, 200, and 350 °C. The relative reflectivity for all DRIFTS spectra is between 1 and 0.5 which indicates that absorbance units are nearly proportional to adsorbate concentration.

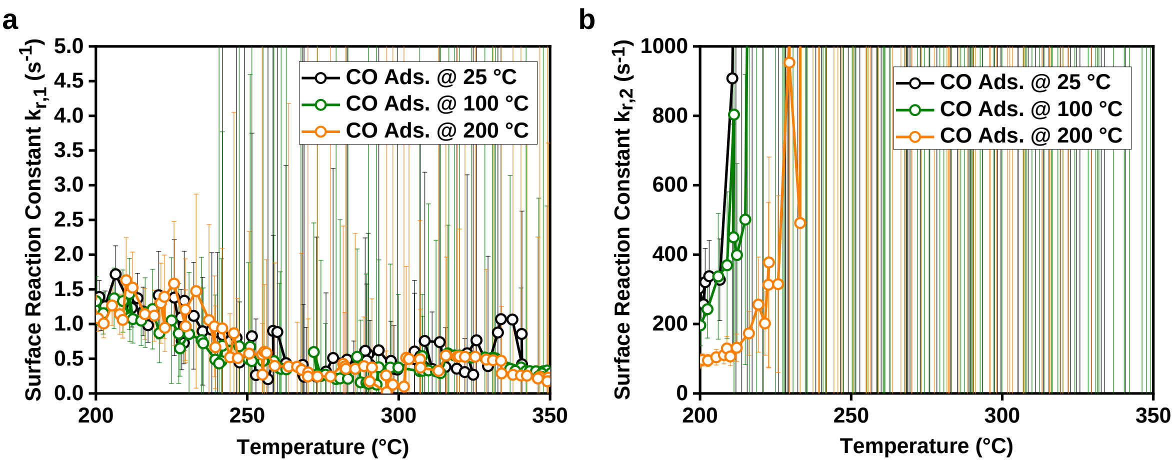

Supplementary Figure 12. Calculated rate constants ($k_{r,1}$ and $k_{r,2}$) for the surface reaction between adsorbed oxygen and CO from the three-pathway kinetic model with 95% confidence intervals overlaid. **a,b**, TPO experiments where CO was adsorbed at 25, 100, and 200 °C with the rate constants zoomed in to the 200–350 °C temperature range.

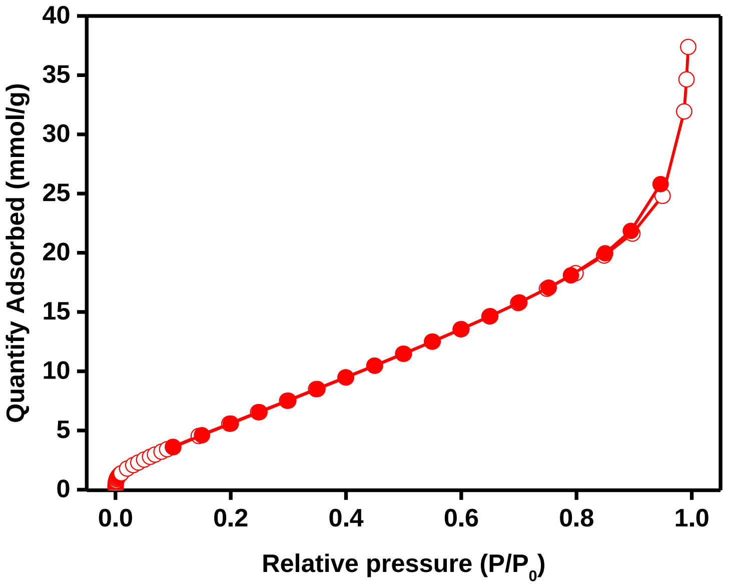

Supplementary Figure 13. N_2 adsorption isotherm plot for SiO_2 support material demonstrating non-nanoporous/macroporous behaviour. BET Measurements were performed using a micrometrics 3flex adsorption analyser. Pressure range: 0 – 1000 mbar, number of data points recorded: 62, equilibration interval: 10 to 45s, standard deviation of fit: 1.735 mmol/g. sample mass: 40 mg. BET Surface area: 514 m^2/g. model: N2 - Tarazona NLDFT, Esf = 30.0K. The sample was degassed at 160 °C under vacuum (10^{-3} torr) for 600 min.

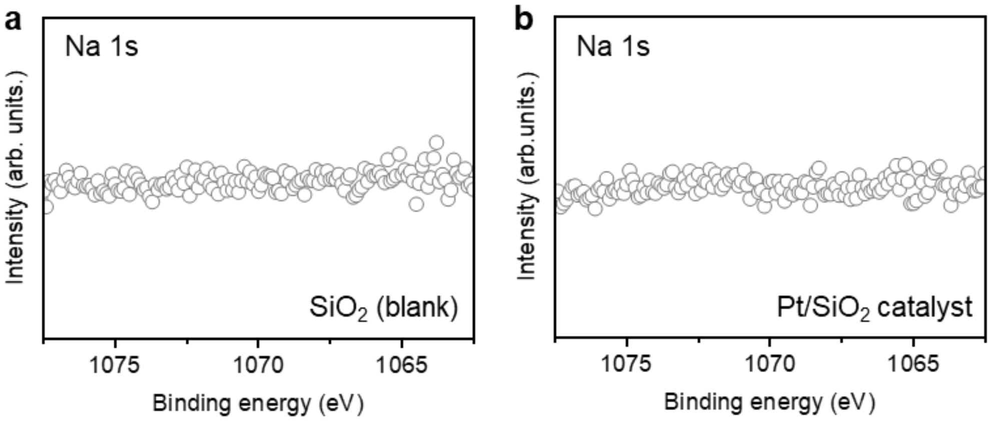

Supplementary Figure 14. XPS measurements for Na 1*s* core-level spectrum. XPS taken over (a) SiO_2 (blank) sample and Pt/SiO_2 catalyst.

IV. Steady-state catalytic measurement *via* home-built flow reactor

The steady-state catalytic reactor used a clear fused quartz tube with a 6.35 mm (1/4 in.) OD and 100 mm overall length, with a 4 mm ID for the catalyst bed and a 2.35 mm ID for the lower half of the reactor. First, a stainless-steel sheathed K-type thermocouple was inserted into the center of the catalyst zone and a quartz wool plug was placed at the joint of the 4 mm ID and 2.35 mm ID tube sections. Next, the catalyst zone consisting of a mixture of inert SiO_2 and catalyst (50-70 mesh sieved grains) was filled up to a 2.6 mm bed height. To ensure we were probing surface reaction kinetics and not internal or external diffusion kinetics, the sieved inert SiO_2 and catalyst (50-70 mesh) was diluted to a 4:1 inert SiO_2 to catalyst volumetric ratio. Lastly, an inert SiO_2 zone was filled up to a total reactor length of 43.3 mm to decrease the volume of the reactor and ensure thermalization of the gas prior to reaching the catalyst.

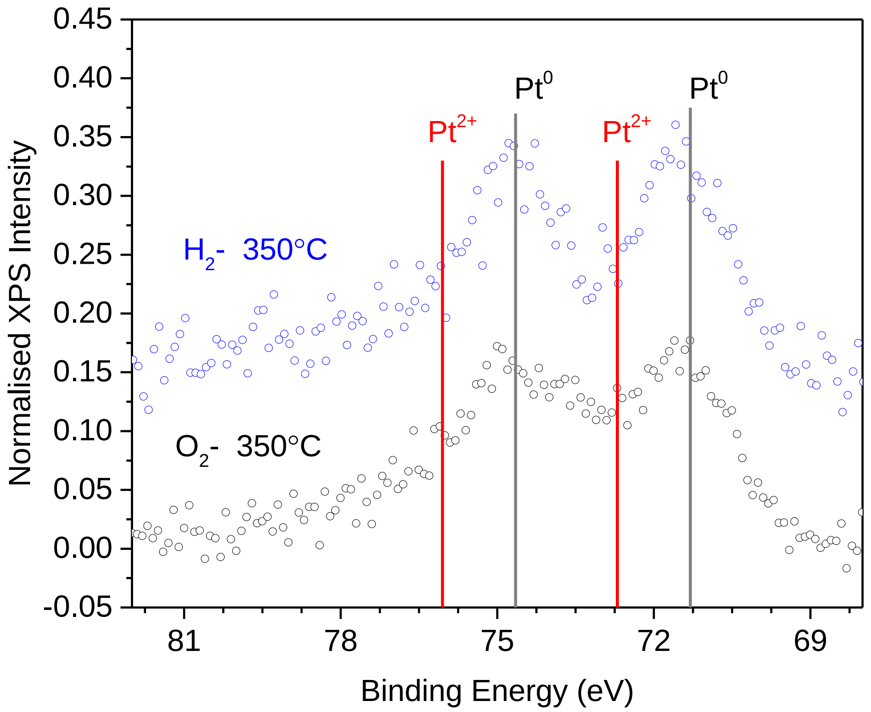

Supplementary Figure 15. XPS measurements for Pt 4*f* core-level spectrum of the Pt/SiO_2 catalyst. XPS spectra taken after O_2 treatment and after a subsequent H_2 treatment at 350 °C.

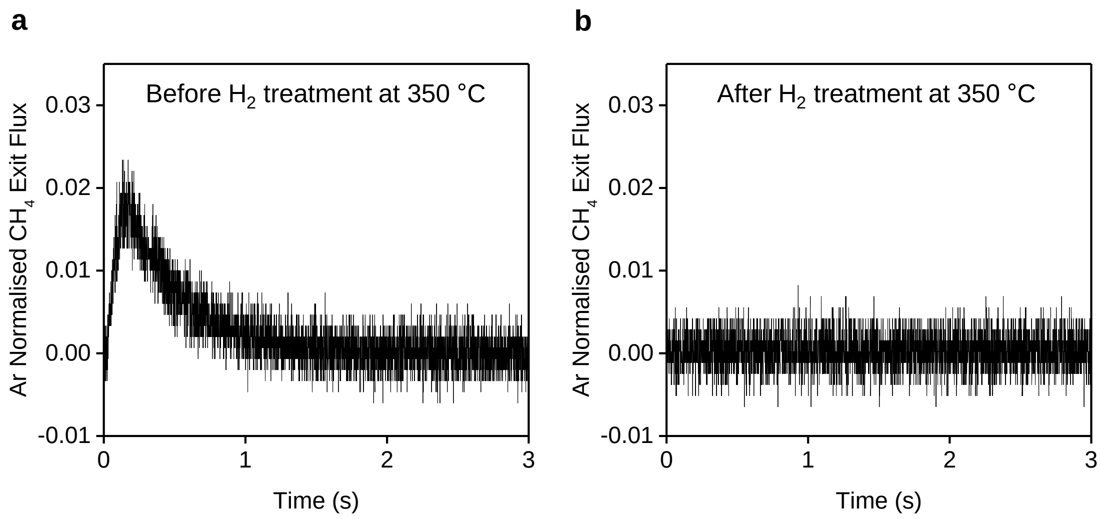

Supplementary Figure 16. Argon normalised exit flux curves of m/z = 16 (CH_4) for a pulse set of 20% of H_2 gas in an inert Ar tracer at 350 °C over oxidised Pt/SiO_2 catalyst (underwent O_2-treatment at 350 °C) (a) before and (b) after H_2 treatment. The lack of CH_4 production indicates that no carbonaceous species can be detected on the catalyst after the combined O_2 and H_2 treatments.

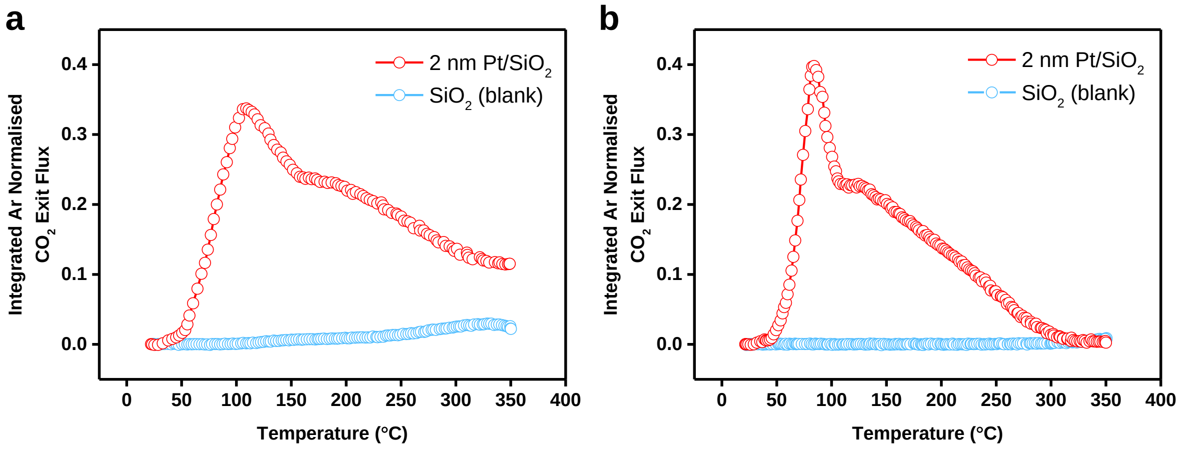

Supplementary Figure 17. **Reactivity tests over a blank catalyst. a**, Temperature-dependent integrated exit flux of m/z = 44 (CO_2) normalised *via* Ar on CO^*-covered Pt/SiO_2 catalyst and SiO_2 (blank) for CO oxidation (6.6% CO 13.4% O_2 gas mixture in an inert Ar tracer) while heating from RT–350 °C at a heating rate of 8 °C/min. **b**, Integrated Ar normalised exit flux of m/z = 44 (CO_2) during TPO experiments on the CO^*-covered Pt/SiO_2 catalyst and SiO_2 (blank) where CO^* was preadsorbed at RT. Then O_2 was repeatedly pulsed over the catalyst while being linearly heated to 350 °C at 8 °C/min.

V. Normalisation/calibration of TAP signal

The integrated exit flux of the Ar tracer is used for normalisation of all pulse experiments. In detail, the exit flux of each reactant gas was divided by that of the Ar tracer in the corresponding pulse set number (*i.e.*, integrated Ar normalised exit flux), which facilitates compensation for the fluctuation of pulse sizes that arises from the valve (~2% variation). For both the isothermal O_2 titration and MZTRT model for TAP experiments, the calibration factor of each gas (*e.g.*, O_2, CO, and CO_2) is required to calculate the conversion of oxygen. Each gas balanced in Ar ($V_X/V_{Ar} = 0.25$; X = O_2, CO, and CO_2) was pulsed into a packed bed microreactor containing only commercial sand (50–70 mesh SiO_2; Sigma-Aldrich). Then the ratio for integrated exit flux of each gas and Ar can be obtained. The calibration factor is obtained by the volume ratio of each gas divided into the ratio for the integrated exit flux of each gas, which was used to calculate the conversion of oxygen in experimental and model calculations.

VI. Absence of an effect for the oxidation of Pt catalyst

We have investigated TAP experiments over the oxidised Pt catalyst which is prepared *via* O_2 treatment at 350 °C. It shows a higher portion of cationic Pt^{2+} species than that of the H_2-treated catalyst in Supplementary Figure 15. As we expected, the oxidised Pt catalyst shows completely different catalytic behaviour compared to metallic Pt in Supplementary Figure 18, indicative of higher catalytic activity at low temperatures (*i.e.*, a lower onset temperature of CO^* conversion) but less amount of CO^* intake. Notably, based on their distinguishable catalytic activity on the oxidised and metallic Pt surface, we can approximate the oxidation state of Pt (*e.g.*, bulk oxidised, partially oxidized, and metallic). To determine when oxidation begins during the CO reaction, we measured the catalytic performance for CO oxidation over the CO^*-covered Pt/SiO_2 for the spent Pt (25–350 °C) and partially spent Pt (25–200 °C). The spent Pt shows similar results to that of the bulk oxidised Pt and the partially spent Pt exhibits similar results to that of the metallic Pt. It is noted that the Pt/SiO_2 catalyst almost consists metallic Pt phase on the surface until 200 °C. Although this is not a direct spectroscopic measurement, it can serve as an indicator to define surface states *via* their distinguishable reactivities. Thus, we can justify our methodology is effective up to at least 200 °C without oxidation effect. We mentioned "When the production of CO_2 is sufficiently low in the TPO experiment (> 200 °C) the signal/noise ratio of the CO_2 exit flux curves significantly decreases, which in turn decreases the confidence in the model fitting, particularly for pathway 2, as shown in Supplementary Fig. 12." in the caption of Figure 7. We think the oxidation of Pt affects the decreases in confidence for the model fitting. Also, Pt catalyst almost certainly gets oxidised at high temperature (> 200 °C) in the TPO experiment, but H_2 treatments were preformed between every experiment. Therefore, we can rule out the oxidation effect in this work.

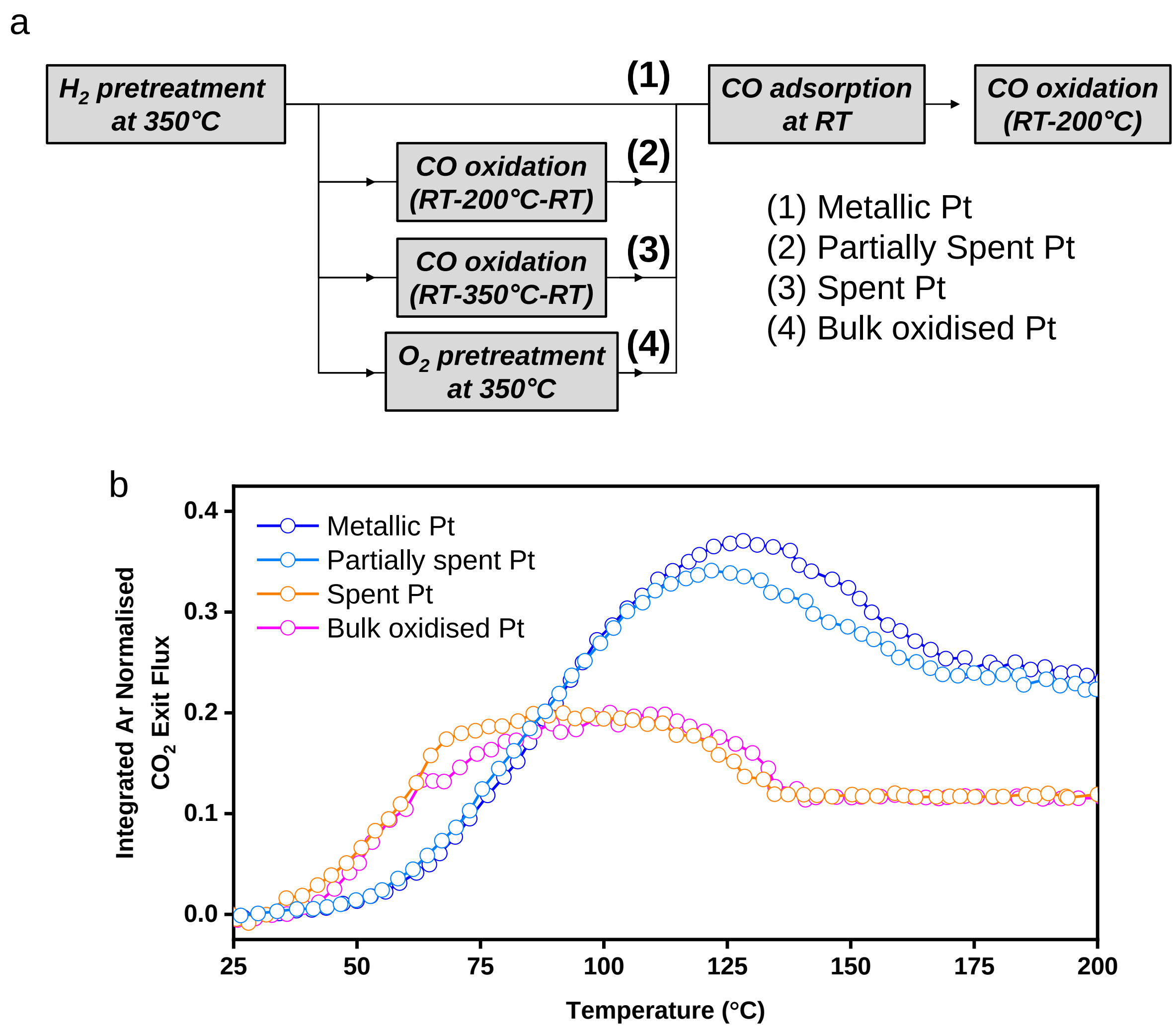

Supplementary Figure 18. Determining catalyst state during reaction conditions. a, Flow chart about the description of four catalysts; the Pt/SiO_2 catalysts prepared by (1) H_2, (2) O_2 pretreatment, CO oxidation (RT–350°C–RT), and CO oxidation (RT–200 °C–RT) are regards to metallic Pt, bulk oxidized Pt, spent Pt, and partially spent Pt catalysts, respectively. **b**, Temperature-dependent integrated exit flux of m/z = 44 (CO_2) normalised *via* Ar on the CO^*-covered Pt/SiO_2 catalysts (the metallic Pt, bulk oxidised Pt, spent Pt, and partially spent Pt) for CO oxidation (6.6% CO 13.4% O_2 gas mixture in an inert Ar tracer); CO^* was preadsorbed at 25 °C while heating from RT–200 °C at a heating rate of 8 °C/min.

Supplementary References

1. Constales, D., Yablonsky, G. S., Marin, G. B. & Gleaves, J. T. Multi-zone TAP-reactors theory and application: I. The global transfer matrix equation. *Chem. Eng. Sci.* **56**, 133–149 (2001).
2. Constales, D., Yablonsky, G. S., Marin, G. B. & Gleaves, J. T. Multi-zone TAP-reactors theory and application. III Multi-response theory and criteria of instantaneousness. *Chem. Eng. Sci.* **59**, 3725–3736 (2004).
3. Constales, D., Yablonsky, G. S., Marin, G. B. & Gleaves, J. T. Multi-zone TAP-reactors theory and application: II. The three-dimensional theory. *Chem. Eng. Sci.* **56**, 1913–1923 (2001).
4. Constales, D., Shekhtman, S. O., Yablonsky, G. S., Marin, G. B. & Gleaves, J. T. Multi-zone TAP-reactors theory and application IV. Ideal and non-ideal boundary conditions. *Chem. Eng. Sci.* **61**, 1878–1891 (2006).
5. Shekhtman, S. O., Yablonsky, G. S., Chen, S. & Gleaves, J. T. Thin-zone TAP-reactor - theory and application. *Chem. Eng. Sci.* **54**, 4371–4378 (1999).
6. Constales, D., Yablonsky, G. S., D'Hooge, D. R., Thybaut, J. W. & Marin, G. B. *Advanced Data Analysis and Modelling in Chemical Engineering*. *Advanced Data Analysis and Modelling in Chemical Engineering* (2016). doi:10.1007/s11144-017-1163-5.
7. Brandão, L., High, E. A., Kim, T.-S. & Reece, C. Simplifying the Temporal Analysis of Products reactor. *Chem. Eng. J.* **478**, 147489 (2023).
8. Kustov, L. M., Ostgard, D. & Sachtler, W. M. H. IR spectroscopic study of Pt/Kl zeolites using adsorption of CO as a molecular probe. *Catal. Lett.* **9**, 121–126 (1991).

9. Kappers, M. J. & Van Der Maas, J. H. Correlation between CO frequency and Pt coordination number. A DRIFT study on supported Pt catalysts. *Catal. Lett.* **10**, 365–373 (1991).
10. Hammer, B., Nielsen, O. H. & Nørskov, J. K. Structure sensitivity in adsorption: CO interaction with stepped and reconstructed Pt surfaces. *Catal. Lett.* **46**, 31–35 (1997).
11. Kale, M. J. & Christopher, P. Utilizing quantitative *in situ* FTIR spectroscopy to identify well-coordinated Pt atoms as the active site for CO oxidation on Al_2O_3-supported Pt catalysts. *ACS Catal.* **6**, 5599–5609 (2016).
12. Avanesian, T. *et al.* Quantitative and atomic-scale view of CO-induced Pt nanoparticle surface reconstruction at saturation coverage via DFT calculations coupled with *in situ* TEM and IR. *J. Am. Chem. Soc.* **139**, 4551–4558 (2017).
13. Kubelka, P. & Munk, F. An article on optics of paint layers. *Z. Tech. Phys.* **12**, 259–274 (1931).
14. Kubelka, P. New contributions to the optics of intensely light-scattering materials part I. *J. Opt. Soc. Am.* **38**, 448 (1948).
15. Sirita, J., Phanichphant, S. & Meunier, F. C. Quantitative analysis of adsorbate concentrations by diffuse reflectance FT-IR. *Anal. Chem.* **79**, 3912–3918 (2007).
16. Matyshak, V. A. & Krylov, O. V. In situ IR spectroscopy of intermediates in heterogeneous oxidative catalysis. *Catal. Today* **25**, 1–87 (1995).
17. Hayden, B. E., Kretzschmar, K., Bradshaw, A. M. & Greenler, R. G. An infrared study of the adsorption of CO on a stepped platinum surface. *Surf. Sci.* **149**, 394–406 (1985).
18. Yoshinobu, J., Tsukahara, N., Yasui, F., Mukai, K. & Yamashita, Y. Lateral displacement by transient mobility in chemisorption of CO on Pt(997). *Phys. Rev. Lett.* **90**, 248301 (2003).

19. Persson, B. N. J., Tüshaus, M. & Bradshaw, A. M. On the nature of dense CO adlayers. *J. Chem. Phys.* **92**, 5034–5046 (1990).
