# Supplementary material for: Interrogating site dependent kinetics over SiO2-supported Pt nanoparticles
Source: Nat Commun. 2024 Mar 7;15:2074. doi: 10.1038/s41467-024-46496-1 (PMC10920675; doi:10.1038/s41467-024-46496-1)
Supplement: Supplementary file 3 — Description of Additional Supplementary Files [file 41467_2024_46496_MOESM3_ESM.pdf]

Description of Additional Supplementary Files

File Name: Supplementary Data 1
Description: Contains an example MATLAB script that performs the MZTRT simulations alongside data for the 25TPO experiment.
